# Noncascading Quadratic Buck-Boost Converter for Photovoltaic Applications

**DOI:** 10.3390/mi12080984

**Published:** 2021-08-19

**Authors:** Rodrigo Loera-Palomo, Jorge A. Morales-Saldaña, Michel Rivero, Carlos Álvarez-Macías, Cesar A. Hernández-Jacobo

**Affiliations:** 1 CONACYT–TecNM/Instituto Tecnológico de La Laguna, Torreón 27000, Mexico; 2 Facultad de Ingeniería, UASLP, San Luis Potosí 78290, Mexico; jmorales@uaslp.mx; 3 Instituto de Investigaciones en Materiales, Unidad Morelia, UNAM, Morelia 58190, Mexico; mrivero@materiales.unam.mx; 4 TecNM/Instituto Tecnológico de La Laguna, Torreón 27000, Mexico; calvarezm@correo.itlalaguna.edu.mx (C.Á.-M.); cahj_22@hotmail.com (C.A.H.-J.)

**Keywords:** switching DC-DC converters, quadratic converter, PV systems

## Abstract

The development of switching converters to perform with the power processing of photovoltaic (PV) applications has been a topic receiving growing interest in recent years. This work presents a nonisolated buck-boost converter with a quadratic voltage conversion gain based on the I–IIA noncascading structure. The converter has a reduced component count and it is formed by a pair of *L*–*C* networks and two active switches, which are operated synchronously to achieve a wide conversion ratio and a quadratic dependence with the duty ratio. Additionally, the analysis using different sources and loads demonstrates the differences in the behavior of the converter, as well as the pertinence of including PV devices (current sources) into the analysis of new switching converter topologies for PV applications. In this work, the voltage conversion ratio, steady-state operating conditions and semiconductor stresses of the proposed converter are discussed in the context of PV applications. The operation of the converter in a PV scenario is verified by experimental results.

## 1. Introduction

In recent decades, the development of power supply systems has exhibited significant growth due to their use in portable systems and equipment, ranging from power supply for the Internet of things (IoT) applications to the energy management of renewable energy sources. In this scenario, the current trend in power electronics systems has required the incorporation of new standards and specifications that must be met, such as higher efficiency, conversion ratios, and power density, amongst others [1]. In these systems, the switching converter is the key of every modern power supply system [2], since it has energy-processing functions aided by suitable control schemes. In this sense, the development of new topologies of switching converters is a topic of interest, as are new structures and/or schemes for the interconnection of converters with high efficiencies. There is also an interest in the application to semiconductor elements with low voltage and/or current stress.

In recent years, a significant number of topologies have been developed that satisfy high transformation ratios, based on isolated and nonisolated schemes, where the implementation is based primarily on the particular application. For example, the main aim in alternating current (AC) power supply systems based on switching converters is to achieve lower total harmonic distortion (THD), a higher power factor, and regulation in the output voltages, as well as higher efficiency [3,4]. In these applications, it is desirable for converters to exhibit galvanic isolation between the output and input of the power system. In the case of direct current (DC) power systems, there are two relevant trends: one is related to the power supply of portable and electronics equipment; the other is related to energy processing and control from renewable sources [5] to develop power systems with high transformation ratios and high efficiency [6,7].

In the emerging renewable energy market, it is a common practice to use switching converters under maximum power point tracking (MPPT) control schemes to obtain the maximum power available in photovoltaic (PV) and/or wind systems. In addition, switching converters must have high conversion ratios, since the voltage levels provided by photovoltaic systems, fuel cells, and/or batteries is a few tens of volts [8,9]. This condition requires systems that allow the processing of energy at the levels of hundreds of volts without or with galvanic isolation capacity.

Currently, switching converters with the capability of increasing or reducing the input voltage for different applications have gained attention. Al–Saffar et al. [4] presented a converter topology based on the interconnection of buck-boost and quadratic buck topologies to obtain high power factor and low output voltage. An approximation for supply systems for light-emitting diode (LED) applications with a high power factor is presented in [10] by Alonso et al. using a quadratic buck-boost converter. The synthesis of a switching converter based on quadratic buck and basic boost topologies was introduced by Nousiainen and Suntio [11,12], where the converter was applied on a photovoltaic system to adapt the energy injected to an inverter. The development of switching converters has led to converters with floating loads [13,14], which restrict their possible applications. In addition, some topologies of circuit synthesis combine the properties of quadratic converters with basic topologies [4,10,11,15,16]. Nowadays, emergent topologies address actual challenges faced by power supply systems for renewable energies, for example, continuous input and output currents, high voltage conversion ratios, common ground path between the input and output ports [14,17,18,19,20,21], and bidirectional power flow properties with quadratic voltage conversion ratios [15,22].

In he technical literature, several solutions exist to obtain switching converters with high voltage conversion ratios. The cascade connection of basic converters is a solution to this problem. Carbajal-Gutiérrez, et al. [23] presented a modeling approach for a quadratic buck converter, where the converter has a high gain to step down the output voltage. In [24], Morales-Saldaña et al. discuss the implementation of a multiloop control scheme in a quadratic boost converter. In [25], Loera-Palomo et al. present a family of quadratic step-down converters based on the noncascade connection of basic switching converters; in [26], a set of converters with a quadratic step-up voltage conversion ratio is introduced. Other quadratic or high-gain converters are presented in [27,28,29,30]. In the context of quadratic converters, in recent years, converters with high buck-boost conversion ratios have been appearing [7,16,17,18,31].

This paper presents a switching converter topology on the basis of the reduced redundant power processing concept, which is based on the interconnection of basic converters in a noncascade structure. The obtained conversion ratio is characterized by a step-up and step-down of the output voltage by a quadratic factor. The proposed converter has a low component count, noninverting output voltage, a common node between the input and output ports, and continuous input current. Additionally, the converter is analyzed with different types of sources and loads where the impact of the PV system in the operation of the converter is investigated.

The remainder of this paper is organized as follows: In Section 2, the proposed converter with wide step-up/down conversion ratio is presented. In addition, a steady-state analysis is given under three scenarios: (1) voltage source–resistive load, (2) PV source–resistive load, and (3) PV source–clamped voltage, to provide insight into the operating conditions of the converter. In Section 3, a representative example is presented to illustrate the main differences in the operating point in steady-state and voltage/current stresses on semiconductor devices. The experimental results of the converter in a PV application are presented in Section 4. Finally, the paper concludes with some remarks in Section 5.

## 2. Proposed Converter

**Figure 1:**
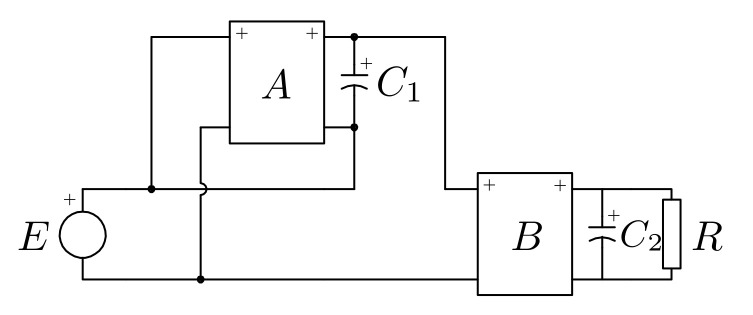
Noncascading I–IIA structure based on the R${}^{2}$P${}^{2}$ concept.

The proposed converter is derived from the noncascading I–IIA structure, which was introduced by Tse et al. in [32,33]. The structure, shown in Figure 1, relates two unidirectional ports (source and load), a bidirectional port (storage element, $C_{1}$), and two general blocks (*A* and *B*), which are formed with basic switching DC-DC cells.

**Figure 2:**
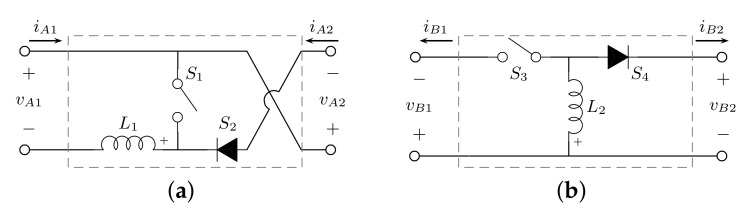
Nonisolated basic switching cells: (**a**) boost; (**b**) buck-boost.

The development of a high-gain switching converter is based on the implementation of two basic switching cells. In block *A*, a boost cell is implemented with inverted output terminals, as shown in Figure 2a. This boost cell changes the voltage polarity in the storage element $C_{1}$, which assures a negative voltage in the input port of block *B*. In turn, a buck-boost cell, as depicted in Figure 2b, provides a positive voltage in the output port of the I–IIA structure.

**Figure 3:**
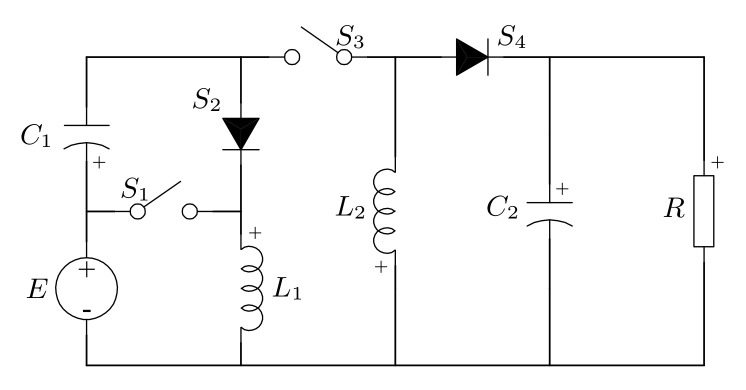
Proposed quadratic buck–boost converter.

The proposed quadratic buck-boost converter is shown in e Figure 3. This topology uses two active switches, $S_{1}$ and $S_{3}$; two passive switches, $S_{2}$ and $S_{4}$; two inductors, $L_{1}$ and $L_{2}$; a storage element, $C_{1}$; and an output capacitor, $C_{2}$. Additionally, *E* represents a voltage source and the load is modeled by a resistance *R*.

Notably, other topologies with high gain or quadratic voltage conversion ratio have been developed under this kind of noncascading structure for PV applications, as discussed in [34,35,36]. Nevertheless, different procedures exist to develop topologies with specific high voltage gain, as stated in [37].

### 2.1. Steady-State Analysis: Voltage Source

In this section, we widen the steady-state analysis of the proposed converter, where the input port of the converter is connected to a voltage supply. In next subsection, the contribution of the PV module and converter is discussed.

The operating modes of the converter are defined by the conditions of the active switches, where the following assumptions are stated:

- The converter operates in continuous conduction mode (CCM).
- The active switches $S_{1}$ and $S_{3}$ operate synchronized to achieve a high voltage gain.
- The operation of the active switches is described by the switching function *q*, and for the passive switches ($S_{2}$ and $S_{4}$) by $\bar{q}=\left(1-q\right)$. They are provided in Equation (1).
  (1) $$\begin{array}{cccc} q & =\left\{\begin{array}{ccc} 1 & \to & 0<t\leq t_{on},\\ 0 & \to & t_{on}<t\leq T_{s}, \end{array}\right. & \;\;\;\;\bar{q} & =\left\{\begin{array}{ccc} 0 & \to & 0<t\leq t_{on},\\ 1 & \to & t_{on}<t\leq T_{s}, \end{array}\right. \end{array}$$
  where $t_{on}$ is the conducting time of the active switches and $T_{s}$ is the switching period.

**Figure 4:**
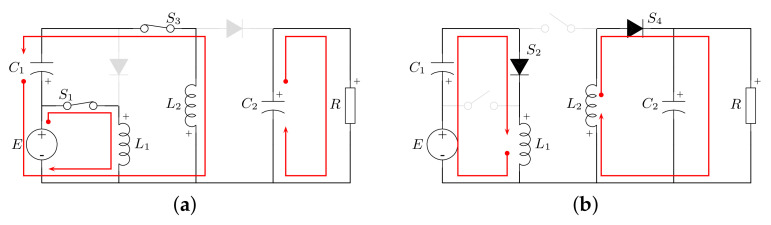
Operating modes of the proposed converter: (**a**) function q=1; (**b**) function q=0.

The operation modes of the converter are described by the on- and off-state of the active switches. During the switch on-state (Figure 4a), inductor $L_{1}$ is connected to the source voltage *E*, whereas inductor $L_{2}$ exhibits voltage $v_{C1}-E$. In this state, capacitor $C_{1}$ transfers energy to inductor $L_{2}$, whereas capacitor $C_{2}$ supports the power demanded by load *R*. In this operating mode, the differential equations that describe the behavior of the system are:

(2) $$\begin{array}{cc} L_{1}\frac{di_{L1}}{dt} & =E,\\ L_{2}\frac{di_{L2}}{dt} & =v_{C1}-E,\\ C_{1}\frac{dv_{C1}}{dt} & =-i_{L2},\\ C_{2}\frac{dv_{C2}}{dt} & =-\frac{v_{C2}}{R}. \end{array}$$

When the switches are in the turn-off state (Figure 4b), inductor $L_{1}$ transfers energy to capacitor $C_{1}$ through diode $S_{2}$, whereas inductor $L_{2}$ supplies energy to conjunct *R*–$C_{2}$ via diode $S_{4}$. The corresponding differential equations are given by:

(3) $$\begin{array}{cc} L_{1}\frac{di_{L1}}{dt} & =E-v_{C1},\\ L_{2}\frac{di_{L2}}{dt} & =-v_{C2},\\ C_{1}\frac{dv_{C1}}{dt} & =i_{L1},\\ C_{2}\frac{dv_{C2}}{dt} & =i_{L2}-\frac{v_{C2}}{R}. \end{array}$$

The set of Equations (2) and (3) defines the voltages at the terminals of the inductors and the currents through the capacitors in one switching period. For these voltages and currents, the principles of inductor volt-second balance and capacitor-charge balance describe a steady-state operation of the converter, which are given by Equations (4) and (5).

(4) $$\begin{array}{cc} \langle v_{L}\left(t\right)\rangle & =0=\frac{1}{T_{s}}\int_{0}^{T_{s}}v_{L}\left(t\right)dt, \end{array}$$

(5) $$\begin{array}{cc} \langle i_{C}\left(t\right)\rangle & =0=\frac{1}{T_{s}}\int_{0}^{T_{s}}i_{C}\left(t\right)dt. \end{array}$$

Using these principles allows us to express the averaged inductor currents and averaged capacitor voltages in the steady-state condition, with the result in Equations (6)–(9).

(6) $$\begin{array}{cc} I_{L1} & =\frac{ED^{3}}{{\left(1-D\right)}^{4}R}, \end{array}$$

(7) $$\begin{array}{cc} I_{L2} & =\frac{ED^{2}}{{\left(1-D\right)}^{3}R}, \end{array}$$

(8) $$\begin{array}{cc} V_{C1} & =\frac{E}{\left(1-D\right)}, \end{array}$$

(9) $$\begin{array}{cc} V_{C2} & =\frac{ED^{2}}{{\left(1-D\right)}^{2}}, \end{array}$$

where *D* is the nominal duty ratio of the converter that defines the on-state time of the active switches by $t_{on}=DT_{s}$. Then, the voltage gain of the converter has a quadratic dependence on the duty ratio, which implies a wide output step-up or step-down voltage conversion characteristic. Therefore, the voltage gain is expressed in Equation (10).

(10) $$M=\frac{V_{C2}}{E}=\frac{D^{2}}{{\left(1-D\right)}^{2}}.$$

It is important to notice that the voltage conversion ratio and the steady-state operating condition are valid only during CCM operation of the converter. In addition, the conversion ratio is positive and the converter presents a common ground between the input and output ports. In this operating mode, two passive networks are formed (Figure 4), in contrast to the discontinuous conduction mode (DCM), where three passive networks are formed, losing the quadratic voltage gain characteristic of the converter.

The boundary conditions between the CCM and DCM operation of the converter are given by the dimensionless parameters as,

(11) $$\begin{array}{cccc} k_{1} & =\frac{2L_{1}}{RT}, & \;\;\;\;\;\;k_{2} & =\frac{2L_{2}}{RT}, \end{array}$$

for inductor $L_{1}$ and $L_{2}$, respectively, for the limit parameter in the following form,

(12) $$\begin{array}{cccc} k_{crit(1)} & =\frac{{\left(1-D\right)}^{4}}{D^{2}}, & \;\;\;\;\;\;k_{crit(2)} & ={\left(1-D\right)}^{2}. \end{array}$$

Then, the converter operates in CCM if it satisfies the criteria in Equation (13).

(13) $$\begin{array}{cccc} k_{1} & >k_{crit(1)}, & \;\;\;\;\;\;k_{2} & >k_{crit(2)}. \end{array}$$

**Figure 5:**
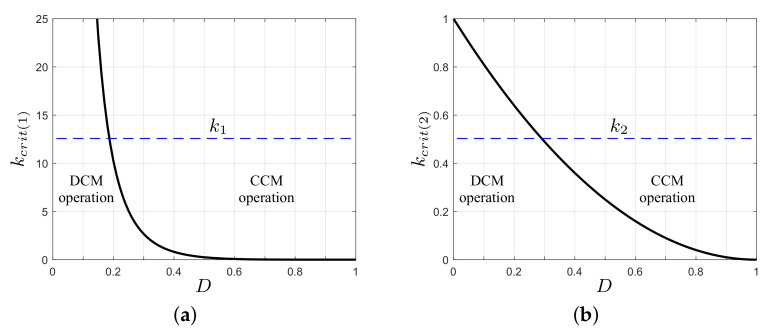
Boundary operation of the proposed converter under parameter variations: (**a**) parameter $k_{crit(1)}$; (**b**) parameter $k_{crit(2)}$.

Figure 5 graphs the limit parameter for each switching cell in the converter, as well as the operative regions depending on the parameters $k_{1}$ and $k_{2}$. In CCM operation, it is easy to show that $k_{2}>1$ satisfies the condition for the switching cell $S_{3}$, $S_{4}$, and $L_{2}$. However, the front-end switching cell ($S_{1}$, $S_{2}$, and $L_{1}$) has an operational limit given by $D^{\prime }<D<1$, where $D^{\prime }$ is the duty ratio that satisfies $k_{1}=k_{crit(1)}$. Therefore, an increase in $k_{1}$ corresponds to an increase in the operational limit of the converter in CCM (reduced value of $D^{\prime }$).

A complete operation of the converter in DCM requires both switching cells to have the same operational limit given by $0<D<D^{\prime }$. The duty ratio $D^{\prime }$ sets the values of $k_{1}$ and $k_{2}$ through the equalities given by Equations (14).

(14) $$\begin{array}{cccc} k_{1} & =k_{crit(1)}\left(D^{\prime }\right), & \;\;\;\;\;\;k_{2} & =k_{crit(2)}\left(D^{\prime }\right). \end{array}$$

Then, the complete voltage conversion ratio of the proposed converter is given by Equation (15). In CCM operation, the voltage gain of the converter depends on the quadratic of the duty ratio, whereas in DCM, it depends on the duty ratio and parameter $k_{1}$.

(15) $$M=\frac{V_{C2}}{E}=\left\{\begin{array}{ccc} \frac{D^{2}}{{\left(1-D\right)}^{2}} & \to & k_{j}>k_{crit(j)}\;\mathrm{with}\;j=1,2,\\ \frac{D}{\sqrt{k_{1}}} & \to & k_{j}<k_{crit(j)}\;\mathrm{with}\;j=1,2. \end{array}\right.$$

In CCM operation, it is assumed that current ripples on the inductors are small, that is, $\Delta I_{L1}\leq \alpha_{1}I_{L1}$ and $\Delta I_{L2}\leq \alpha_{2}I_{L2}$, where $0<\alpha_{j}\leq 1$. In this sense, the inductances required to keep the ripples values below a given threshold are provided by Equation (16).

(16) $$\begin{array}{cccc} L_{1} & =\frac{{\left(1-D\right)}^{4}RT}{\alpha_{1}D^{2}}, & \;\;\;\;\;\;L_{2} & =\frac{{\left(1-D\right)}^{2}RT}{\alpha_{2}}. \end{array}$$

### 2.2. Steady-State Analysis: Current Source

**Figure 6:**
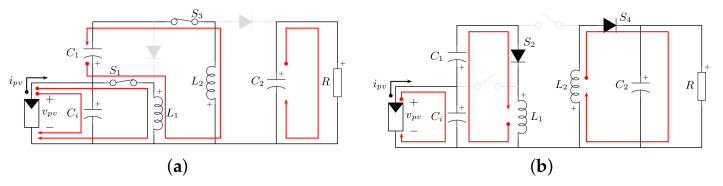
Operating modes of the proposed converter using a PV module. (**a**) Function q=1. (**b**) Function q=0.

The consideration of a current source in the converter is related to PV applications, since a PV module/array is modeled as a current source. As a consequence, the voltage source of the converter in Figure 3 is changed by a PV module and a coupling capacitor $C_{i}$. In this system, the switching function in Equation (1) describes the operation of the active and passive switches. Figure 6 shows the resulting networks by the operation of the converter according to this switching function.

In PV applications, during the switch on-state (Figure 6a), the inductor $L_{1}$ is connected to coupling capacitor $C_{i}$ and exhibits voltage $v_{Ci}$, whereas inductor $L_{2}$ exhibits voltage $v_{C1}-v_{Ci}$. In this scenario, both inductors are in charge mode. The PV module transfers energy to capacitor $C_{i}$; in turn, capacitor $C_{1}$ transfers energy to inductor $L_{2}$ and $C_{2}$ supports the load power demand. Equations (17) describe the behavior of the system in this state.

(17) $$\begin{array}{cc} L_{1}\frac{di_{L1}}{dt} & =v_{Ci},\\ L_{2}\frac{di_{L2}}{dt} & =v_{C1}-v_{Ci},\\ C_{i}\frac{dv_{Ci}}{dt} & =i_{pv}-i_{L1}+i_{L2},\\ C_{1}\frac{dv_{C1}}{dt} & =-i_{L2},\\ C_{2}\frac{dv_{C2}}{dt} & =-\frac{v_{C2}}{R}. \end{array}$$

Conversely, in the turn-off state of the active switches (Figure 6b), inductor $L_{1}$ and capacitor $C_{i}$ transfer energy to capacitor $C_{1}$, while inductor $L_{2}$ supplies energy to conjunct *R*–$C_{2}$. The differential equations in this condition are given by Equations (18).

(18) $$\begin{array}{cc} L_{1}\frac{di_{L1}}{dt} & =v_{Ci}-v_{C1},\\ L_{2}\frac{di_{L2}}{dt} & =-v_{C2},\\ C_{i}\frac{dv_{Ci}}{dt} & =i_{pv}-i_{L1},\\ C_{1}\frac{dv_{C1}}{dt} & =i_{L1},\\ C_{2}\frac{dv_{C2}}{dt} & =i_{L2}-\frac{v_{C2}}{R}. \end{array}$$

The above expressions define the voltages at the terminals of the inductors and the currents through the capacitors in one switching period. The steady-state condition of the converter can by obtained by using the principles of volt-second and charge balance, which result in Equations (19)–(23).

(19) $$\begin{array}{cc} I_{L1} & =\frac{I_{pv}}{D}, \end{array}$$

(20) $$\begin{array}{cc} I_{L2} & =\frac{\left(1-D\right)I_{pv}}{D^{2}}, \end{array}$$

(21) $$\begin{array}{cc} V_{Ci} & =\frac{{\left(1-D\right)}^{4}I_{pv}R}{D^{4}}, \end{array}$$

(22) $$\begin{array}{cc} V_{C1} & =\frac{{\left(1-D\right)}^{3}I_{pv}R}{D^{4}}, \end{array}$$

(23) $$\begin{array}{cc} V_{C2} & =\frac{{\left(1-D\right)}^{2}I_{pv}R}{D^{2}}. \end{array}$$

The voltage conversion gain of the converter can be obtained using Expressions (21) and (23), where the relation between the output voltage and the coupling capacitor voltage maintain a quadratic dependence, given by Equation (24).

(24) $$M=\frac{V_{C2}}{V_{Ci}}=\frac{D^{2}}{{\left(1-D\right)}^{2}}.$$

The power generated by the PV module/array depends on weather conditions, that is, incident solar irradiance and temperature. In addition, the voltage at the terminals permits the adjustment of the power supplied by the PV module. Since the maximum power of a PV module is related to a specific voltage, Equation (21) shows that the voltage in the PV module can be selected by using the duty ratio signal of the converter, that is

(25) $$D=\frac{{\left[I_{pv}R\right]}^{\frac{1}{4}}}{{\left[I_{pv}R\right]}^{\frac{1}{4}}+{\left[V_{Ci}\right]}^{\frac{1}{4}}}.$$

Finally, current $I_{o}$ injected to load (*R*) is given by Equation (26). This implies that the output voltage of converter ($V_{C2}$) is not fixed and it depends on the duty ratio, the generated current by the PV module, and the value of load *R*.

(26) $$I_{o}=\frac{{\left(1-D\right)}^{2}I_{pv}}{D^{2}}=\frac{I_{pv}}{M}.$$

The inequality (13), which is the boundary condition between the CCM and DCM operation of the converter, holds independently of the PV application. Additionaly, the inductance values are given by Expressions (16).

**Figure 7:**
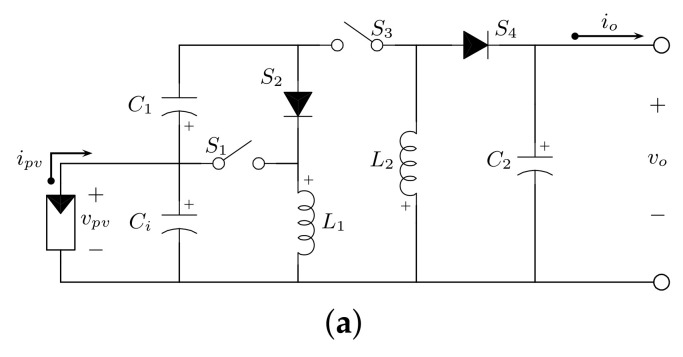

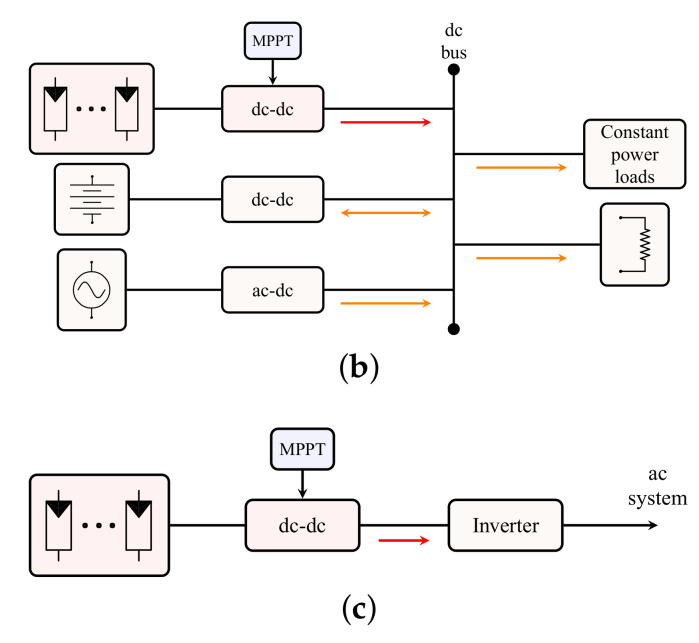
Structure of PV applications. (**a**) Proposed converter. (**b**) DC network application. (**c**) Grid-connected PV system.

PV systems can be applied in different ways; however, there are some applications in which the output voltage of the power converter is clamped. Figure 7a shows the proposed converter topology, where the output power condition is given in terms of the output current and output voltage. Figure 7b shows the implementation of the PV system in a DC microgrid, where the output voltage of the converter corresponds to the bus voltage of the microgrid. Finally, Figure 7c shows a grid-connected application, where the output voltage of the converter is clamped or regulated by the inverter (DC-AC converter). In this kind of scenario, the operating point of the converter and the semiconductor stresses are different than those presented by a pure resistive load.

A similar analysis applied to the converter topology in Figure 7a shows that the steady-state condition of the converter results in Equations (27)–(31).

(27) $$\begin{array}{cc} I_{L1} & =\frac{I_{pv}}{D}, \end{array}$$

(28) $$\begin{array}{cc} I_{L2} & =\frac{\left(1-D\right)I_{pv}}{D^{2}}, \end{array}$$

(29) $$\begin{array}{cc} V_{Ci} & =\frac{{\left(1-D\right)}^{2}v_{o}}{D^{2}}, \end{array}$$

(30) $$\begin{array}{cc} V_{C1} & =\frac{V_{Ci}}{\left(1-D\right)}, \end{array}$$

(31) $$\begin{array}{cc} V_{C2} & =v_{o}. \end{array}$$

Equation (29) shows that the voltage conversion ratio ($M=V_{C2}/V_{Ci}$) of the converter maintains a quadratic dependence on the duty ratio. Additionally, to achieve a specific voltage at the terminals of the PV module requires the duty ratio to be

(32) $$D=\frac{\sqrt{V_{C2}}}{\sqrt{V_{C2}}+\sqrt{V_{Ci}}}.$$

In addition, the current injected to the next system $I_{o}$ corresponds to the expression given in Equation (26).

### 2.3. Semiconductor Stress

An aspect of interest in designing renewable-energy-based power systems is the stress level of the semiconductor elements. As such, the expressions for the current and voltage are derived.

For the proposed converter, the expressions of voltage and current stress on the semiconductor devices are presented in Table 1 for the three applications analyzed in this work. $V_{s1}$ ($I_{s1}$) and $V_{s3}$ ($I_{s3}$) are the voltage (current) stress on the active switches $S_{1}$ and $S_{3}$, respectively; whereas $V_{s2}$ ($I_{s2}$) and $V_{s4}$ ($I_{s4}$) are the voltage (current) stress on the passive switches (diodes) $S_{2}$ and $S_{4}$, respectively.

It is important to notice that the voltage and current stress on semiconductor devices depend on the duty ratio of the converter. However, depending on the external elements (sources and loads) connected to the converter, the voltage and current stress might differ. The main difference in the stress on a semiconductor is related to the PV application, where a PV module has a particular behavior depending on the weather conditions and the operation point.

### 2.4. Dynamic Model

A state-space averaged model of converters is a key aspect in the control of power electronic systems. In this study, averaged models were developed based on common techniques to complement the study of the proposed converter under previously mentioned applications. The resulting model, when a voltage source is used in the input port, is given by Equations (33).

(33) $$\begin{array}{cc} L_{1}\frac{di_{L1}}{dt} & =E-\left(1-d\right)v_{C1}-R_{p1}i_{L1},\\ L_{2}\frac{di_{L2}}{dt} & =d\left(v_{C1}-E\right)-\left(1-d\right)v_{C2}-R_{p2}i_{L2},\\ C_{1}\frac{dv_{C1}}{dt} & =\left(1-d\right)i_{L1}-di_{L2},\\ C_{2}\frac{dv_{C2}}{dt} & =\left(1-d\right)i_{L2}-\frac{v_{C2}}{R}. \end{array}$$

The averaged model, when a PV module with a coupling capacitor is used in the input port and a resistive load in the output port, results in

(34) $$\begin{array}{cc} L_{1}\frac{di_{L1}}{dt} & =v_{Ci}-\left(1-d\right)v_{C1}-R_{p1}i_{L1},\\ L_{2}\frac{di_{L2}}{dt} & =d\left(v_{C1}-v_{Ci}\right)-\left(1-d\right)v_{C2}-R_{p2}i_{L2},\\ C_{1}\frac{dv_{C1}}{dt} & =\left(1-d\right)i_{L1}-di_{L2},\\ C_{2}\frac{dv_{C2}}{dt} & =\left(1-d\right)i_{L2}-\frac{v_{C2}}{R},\\ C_{i}\frac{dv_{Ci}}{dt} & =i_{pv}-i_{L1}+di_{L2}. \end{array}$$

Finally, the averaged model of the converter in a PV application connected to a DC voltage system corresponds to

(35) $$\begin{array}{cc} L_{1}\frac{di_{L1}}{dt} & =v_{Ci}-\left(1-d\right)v_{C1}-R_{p1}i_{L1},\\ L_{2}\frac{di_{L2}}{dt} & =d\left(v_{C1}-v_{Ci}\right)-\left(1-d\right)v_{C2}-R_{p2}i_{L2},\\ C_{1}\frac{dv_{C1}}{dt} & =\left(1-d\right)i_{L1}-di_{L2},\\ C_{2}\frac{dv_{C2}}{dt} & =\left(1-d\right)i_{L2}-i_{o},\\ C_{i}\frac{dv_{Ci}}{dt} & =i_{pv}-i_{L1}+di_{L2}. \end{array}$$

In the above models, d(t) represents the averaged duty ratio; $R_{pn}$, with n=1,2, represents the parasitic resistances in the converter in Equation (36).

(36) $$R_{pn}=R_{Ln}+R_{Mn}d+R_{Dn}\left(1-d\right),$$

where $R_{Ln}$ is the resistance in the inductor, and $R_{Mn}$ and $R_{Dn}$ are the on-resistances of the active and passive switches, respectively.

### 2.5. Comparison

In the literature, several solutions of quadratic buck-boost converters for photovoltaic applications have been published. The properties of these converters are summarized in Table 2, where the proposed converted is included for the sake of comparison. Note that all converters present a quadratic gain.

The topologies presented in [7,16,18] share a common characteristic, continuous input and output current, but this feature is penalized by the component number, since three inductors and three capacitors are required; additionally, [16] increased the semiconductors by two with respect to the other solutions. The proposed converter partially fulfills this requirement, where an output filter can be added to ensure a continuous output current, increasing the number of components.

In semiconductor devices, the voltage stress in previous topologies and in the proposed converter is similar when a voltage source and a resistive load are used. An interesting point regarding the previous topologies is their use or applicability in PV applications. Those works considered a voltage source and a resistive load that do not represent the operation of the converter in a PV application. Conversely, in this paper, we show that a PV source with a resistive load or clamped output voltage changes the voltage stress on semiconductor devices.

## 3. Numerical Analysis and Discussion

In this section, a numerical analysis is described to better demonstrate the performance of the proposed converter under different operating conditions, as well as to evaluate the electrical stress on the semiconductor devices. In a PV application scenario, we used a photovoltaic module LUXEN LNSA-160P (Luxen Solar Energy Co., Suzhou, China). The PV module (listed in Table 3) was characterized under the following conditions: 814 W/m${}^{2}$ solar irradiance, ambient temperature 29.2${}^{\circ }$C, and operating temperature of 63.13 ${}^{\circ }$C.

Now, we turn our attention to the three studied cases (voltage converter application, PV application with resistive load, and PV application with clamped output voltage) under equal nominal operating conditions, but taking into account the maximum power point (MPP) of the PV module. The specifications of the proposed converter are: nominal supplied voltage E=14.01 V, nominal PV current $I_{pv}=7.413$ A, output port voltage of 56 V, nominal power of 103.9 W, and resistive load R=30.183Ω. Using the aforementioned specifications allowed us to determine the nominal operating point of the converter in each application, described in Table 4.

**Figure 8:**
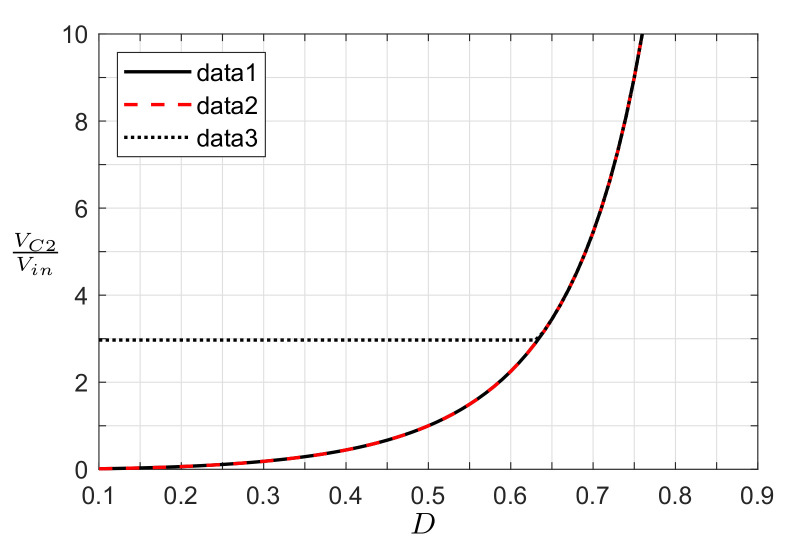
Voltage conversion gain (data 1: voltage converter appl; data 2: PV appl, resistive load; data 3: PV appl, clamped voltage).

From the previous analysis, we observed that the proposed converter presents a buck-boost voltage conversion ratio that depends on the square of the duty ratio. Figure 8 plots the converter gain as a function of the duty ratio for the three application cases. In this plot, the voltage/PV applications with resistive load present the same voltage gain;,whereas the PV application with clamped voltage has a constant gain for D≤0.633, and then it exhibits the same gain. This behavior is due to the operating characteristics of the PV module, where the voltage at the terminals has a limited range, given by $0<V_{pv}<V_{oc}$, with $V_{oc}$ being the maximum voltage in the PV module (zero power supply). Expression (32) shows that the point of constant gain can change depending on the characteristics/technology of the PV module and the selected output voltage.

**Figure 9:**
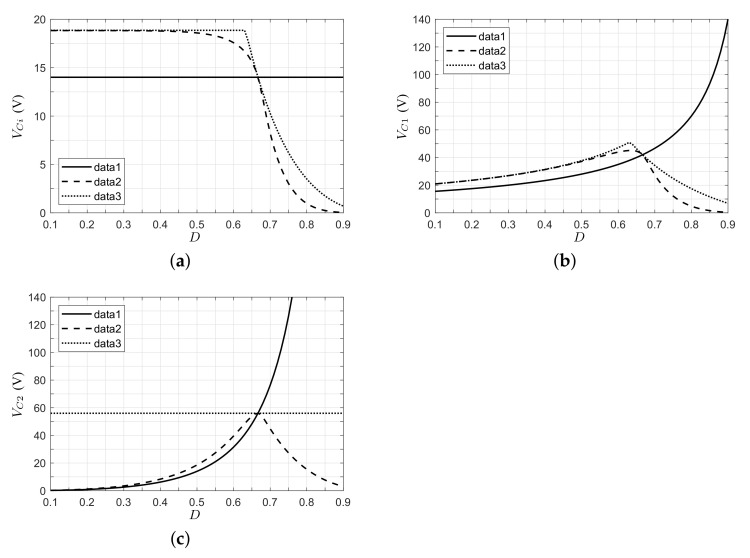
Capacitor voltages in the steady state of the proposed converter (data 1: voltage converter appl; data 2: PV appl, resistive load; data 3: PV appl, clamped voltage). (**a**) Input port. (**b**) Capacitor $C_{1}$. (**c**) Output port.

The converter presents the same voltage gain (or partially) in different application cases; however, the voltages and currents in the converter are different due to the PV module’s behavior. Figure 9 shows the voltages in the capacitors as a function of the duty ratio. In a voltage converter application (data 1, solid line), it is evident that supply voltage is constant (ideal voltage source); for this reason, when the duty cycle increases, the voltage in capacitors $C_{1}$ and $C_{2}$ (output port) increases too. A PV application with a resistive load (data 2, dashed line) behaves differently. In this scenario, for low duty ratios, the voltage at the terminals of the PV module is equal to $V_{oc}$, and when the duty cycle increases, the voltage $V_{pv}$ reduces. This finding implies that the voltage in the PV module can be controlled by the duty cycle signal, as well as the power delivered by the PV module. Additionally, the output voltage increases until the maximum PV power (nominal operating point) is reached; after this point, the output voltage decreases as the voltage and current in the PV module tend to zero. Finally, in the PV application with clamped output voltage (data 3, dotted line), $V_{pv}$ is constant (equal to $V_{oc}$) at low duty cycles since the voltage gain of the converter is lower than the relation $V_{C2}/V_{oc}$. When the duty cycle increases and reaches an appropriate voltage converter gain ($M\left(D\right)=V_{C2}/V_{oc}$), a further increase in the duty ratio implies a reduction in $V_{pv}$, where the voltage in the PV module can be controlled by the converter. Then, the operative region of the converter depends on the gain of the converter, clamped output voltage, and voltage range ($0<V_{pv}<V_{oc}$) of the PV module.

**Figure 10:**
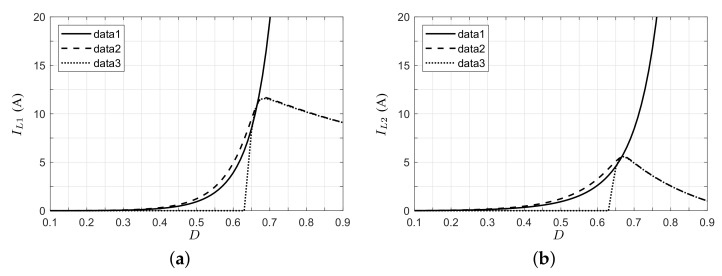
Inductor currents in the steady state of the proposed converter (data 1: voltage converter appl; data 2: PV appl, resistive load; data 3: PV appl, clamped voltage). (**a**) Inductor $L_{1}$. (**b**) Inductor $L_{2}$.

Figure 10 shows the inductor currents of the converter for the three cases analyzed. Here, it can be observed that the inductor currents in the voltage converter application increase with duty cycle, and these currents are higher above the nominal operating point due to the increase in the power demand. In the case of a PV with a resistive load, the inductor currents increase until the nominal operating point is reached; after, the current decreases as a function of the duty cycle and the current $I_{pv}$, which tends to the maximum current $I_{sc}$. In the case where the output voltage is clamped, the inductor currents are zero until the converter reaches an appropriate voltage gain, after which the currents increase in value.

**Figure 11:**
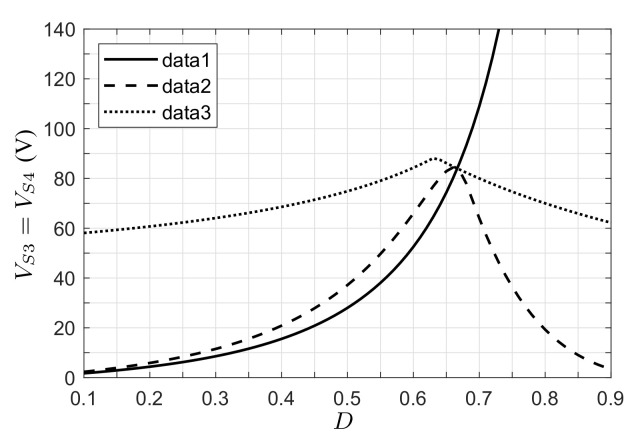
Voltage stress on semiconductors $S_{3}$ and $S_{4}$ (data 1: voltage converter appl; data 2: PV appl-, resistive load; data 3: PV appl, clamped voltage).

Now, considering the voltage and current stress on semiconductor devices, and based on the expressions in Table 1, it can be shown that the voltage and current stress on active switch $S_{1}$ and passive switch $S_{2}$ are related to voltage $V_{C1}$ and current $I_{L1}$; the current stress on semiconductors $S_{3}$ and $S_{4}$ is related to current $I_{L2}$. For semiconductors $S_{1}$ and $S_{2}$, the voltage stress is equal ($V_{S1}=V_{S2}=V_{C1}$), as shown in Figure 9b. In this plot, the PV application presents a greater voltage stress than the voltage source application, which can be explained by the voltage of the PV module being near the $V_{oc}$ value. After this value, the voltage stress reduces as the voltage of the PV module tends to zero. In turn, the current stresses ($I_{S1}=I_{S2}=I_{L1}$ and $I_{S3}=I_{S4}=I_{L2}$) for the clamped voltage application are lower than for the voltage source application, since the PV module current approaches zero when the PV module voltage is near the $V_{oc}$ value (Figure 10). After the maximum power point of the PV module, the current stress is limited since the PV module current is practically constant. Finally, the voltage stress on semiconductors $S_{3}$ and $S_{4}$ is plotted in Figure 11. Here, it can be observed that the voltage stress is greater for the output voltage clamped case than for the PV application with load resistance case.

## 4. Experimental Results

**Figure 12:**
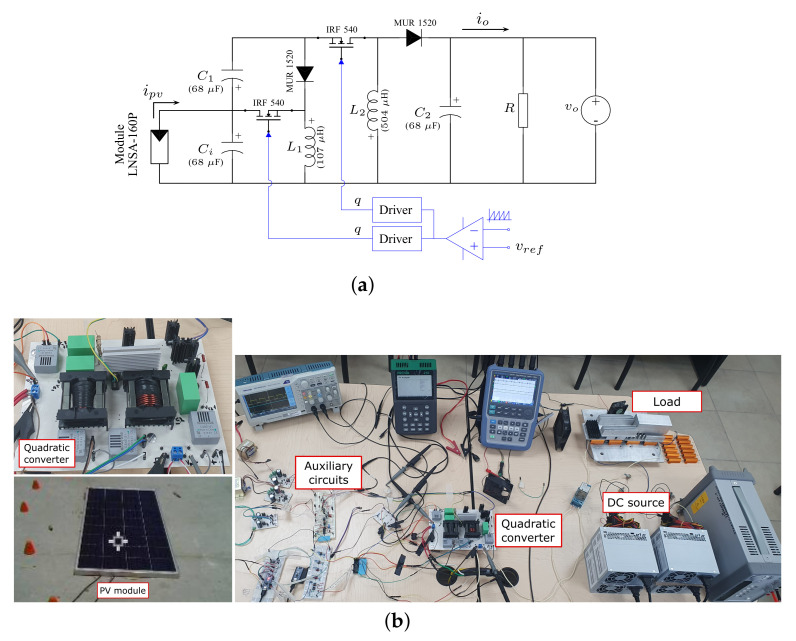
Experimental prototype of step-up/down quadratic converter. (**a**) Circuit diagram. (**b**) Experimental setup.

To verify the behavior of the proposed converter, we conducted an experimental text. The test circuit is shown in Figure 12. The PV module LUXEN LNSA-160P has specifications at standard test conditions (STCs) of 160 W, 18.30 V, and 8.75 A at the maximum power point. The source $v_{o}$ fixes the voltage to a desired value. Additionally, since the voltage source $v_{o}$ does not accept input currents, a load *R* was added to dissipate the power generated by the PV module. Finally, a switching frequency of 50 kHz was used for the proposed converter.

**Figure 13:**
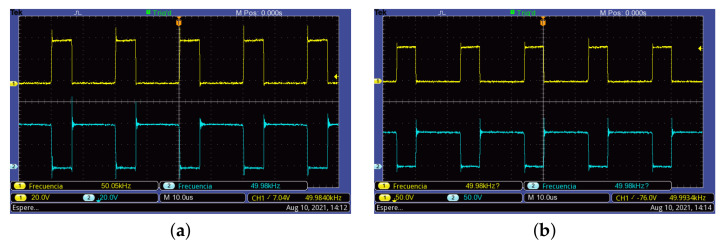
Switching device voltages. (**a**) Active switch $S_{1}$ (**Top**) and diode $S_{2}$ (**Bottom**). (**b**) Active switch $S_{3}$ (**Top**) and diode $S_{4}$ (**Bottom**).

**Figure 14:**
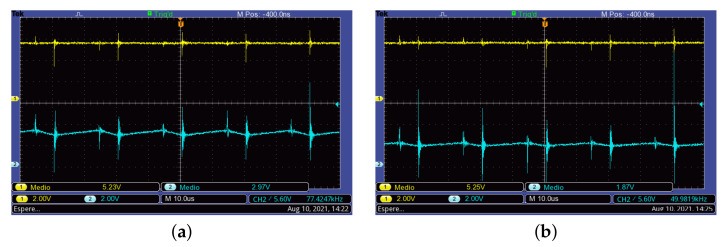
Inductor and PV module currents. (**a**) PV module (**Top**) and inductor $L_{1}$ (**Bottom**). (**b**) PV module (**Top**) and inductor $L_{2}$ (**Bottom**).

**Figure 15:**
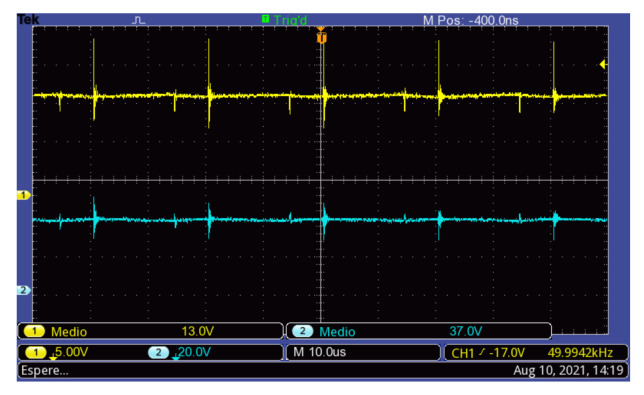
Capacitor voltages: capacitor $C_{i}$ (**Top**) and capacitor $C_{1}$ (**Bottom**).

The resulting waveforms of currents and voltages from the operation of the proposed converter are shown in Figure 13, Figure 14 and Figure 15. This test was performed with a clamped output voltage of $v_{o}=56$ V, an incident solar radiation of 912 W/m${}^{2}$, and a PV module temperature of 67 ${}^{\circ }$C. Figure 13 shows the voltage at the terminals of each switching devices, where it can be observed that active switches operate synchronously, whereas passive switches (diodes) have a complementary operation. In all cases, well-defined transitions are observed.

Figure 14 shows the waveforms of the current of the PV module and the inductor currents. Here, it can be observed that both inductor currents increase linearly when the active switches are turned on, and the current decreases when the switches are turned off. The measured average current in each element is $I_{pv}=7.622$ A, $I_{L1}=10.8$ A, and $I_{L2}=4.53$ A, whose values are consistent with those obtained from Expressions (27) and (28).

Finally, the waveforms of the voltages in the switching converter capacitor $C_{i}$ (input port) and capacitor $C_{1}$ are shown in Figure 15. In this condition, the voltage at the terminals of the PV module is $V_{pv}=13$ V, which is lower than the open-circuit voltage $V_{oc}=19.18$ V, whereas the voltage in the capacitor $C_{1}$ is $V_{C1}=37$ V.

**Figure 16:**
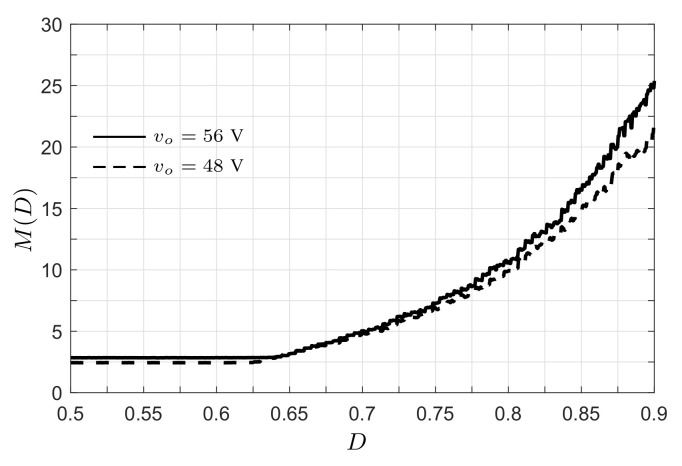
Experimental voltage conversion ratio using $v_{o}=56$ V (solid line) and $v_{o}=48$ V (dashed line).

The experimental voltage conversion ratio ($M=V_{o}/V_{Ci}$) of the proposed converter is shown in Figure 16. In the test, the output voltage was maintained constant (test 1: $V_{o}$ = 56 V, and test 2: $V_{o}$ = 48 V), whereas the duty ratio was changed step-by-step from 0.5 to 0.9. At the start of the test, the conversion ratio was constant since the voltage at terminals of the PV module is equal to its open-circuit voltage ($V_{pv}=19.9$ V). When the duty ratio increases, the converter forces the reduction in the PV module voltage, increasing the conversion ratio of the converter. The maximum conversion ratio at D=0.9 is M=25.64 for $V_{o}=56$ V, and M=21.87 for $V_{o}=48$ V.

**Figure 17:**
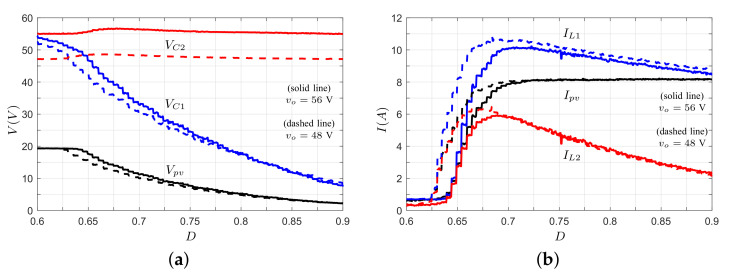
Currents and voltages in the converter. (**a**) Voltage at the terminal of the PV module: capacitor $C_{1}$ and capacitor $C_{2}$. (**b**) Current generated by the PV module: inductor $L_{1}$ and inductor $L_{2}$.

Figure 17 shows the main voltages and currents of the converter when the duty cycle is varied from 0.6 to 0.9. In Figure 17a, the PV module voltage is reduced when the duty ratio increases; therefore, the PV module voltage can be controlled through the duty ratio, as can the power delivered by the PV module. It can be observed in Figure 17b that the PV module current increases until it reaches a constant current region. Here, a further increase in the duty ratio implies that voltages $V_{pv}$ and $V_{C1}$ tend to zero, whereas the current $I_{pv}$ tends to the short-circuit current, which is consistent with the operation of the PV module.

**Figure 18:**
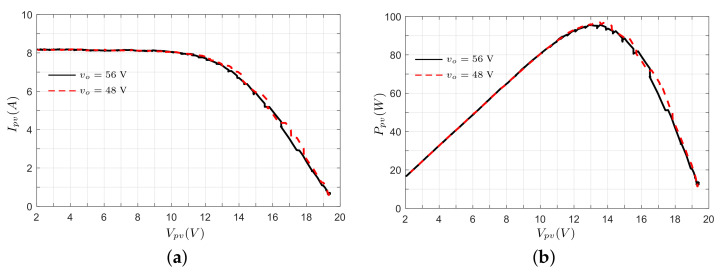
Experimental results in the converter’s input port. (**a**) I–V curve. (**b**) P–V curve.

Figure 18 shows the power–voltage (P–V) and current–voltage (I–V) curves of the PV module, which were built with the measured input current and voltage of the converter. In Figure 18a, it can be observed that an increase in the duty ratio increases the current $I_{pv}$, whereas the voltage $V_{pv}$ decreases. This behavior continues until the PV module enters the constant current region. Figure 18b presents the behavior of the power delivered in the PV module when the duty ratio is varied. In this test, ia maximum power of 95.61 W is obtained at D=0.68, where $I_{pv}=7.054$ A and $V_{pv}=13.55$ V.

**Figure 19:**
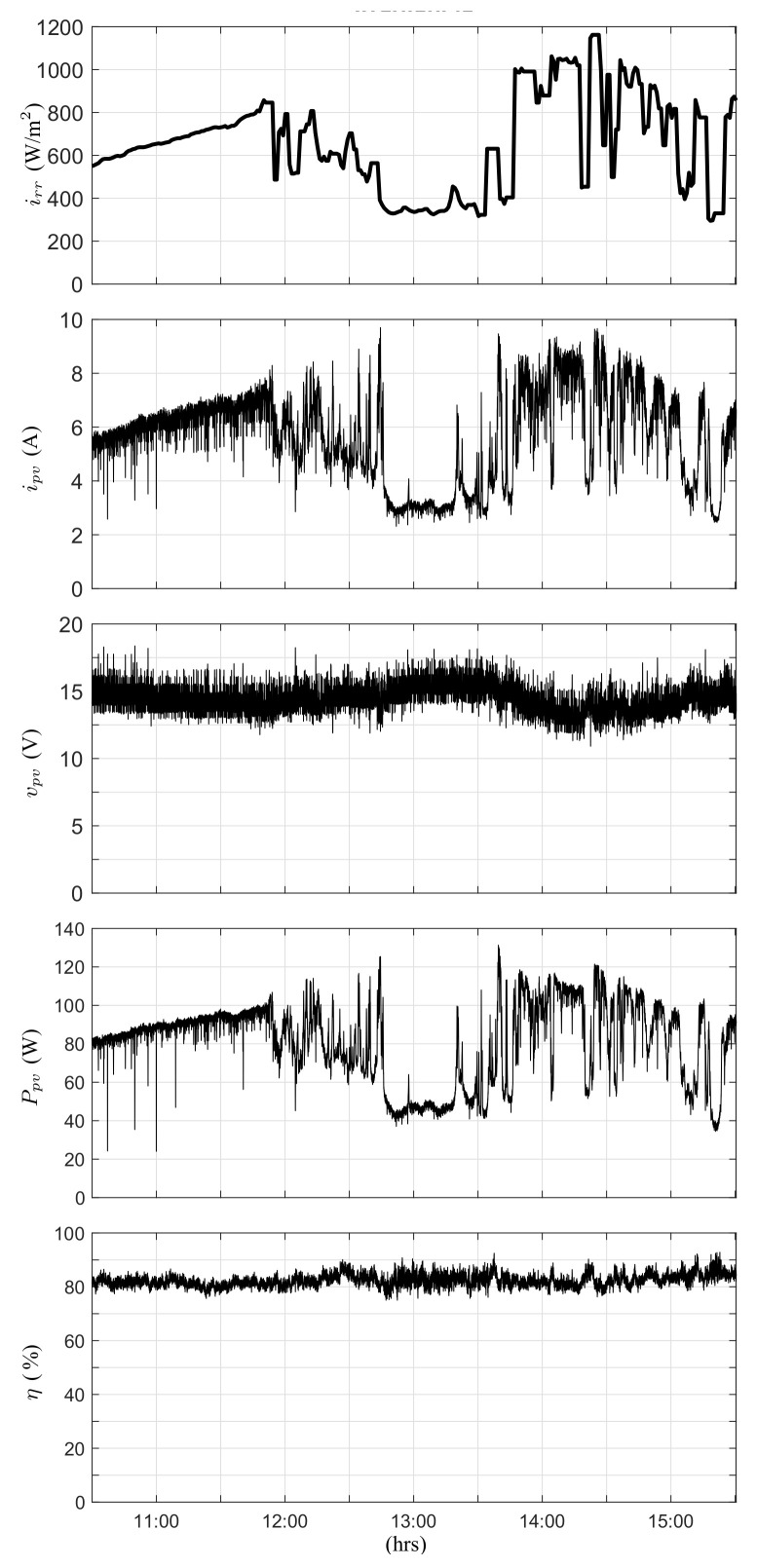
Time responses of the switching converter with an MPPT perturb and observe algorithm. (**Top**) to (**bottom**): Measured global solar irradiance, generated current of the PV module, voltage at the terminals, power developed by the PV module, and efficiency (partially cloudy day).

Then, the proposed converter was tested in a scenario to determine the maximum power of the PV module. In this test, the duty cycle of the converter was determined by a perturb and observe (P&O) MPPT algorithm. Figure 19 shows the principal measurements that confirm the operation of the converter in a day with partially cloudy conditions. Global solar irradiance shows a significant variability due to clouds, which is strongly correlated with the current and power developed. The voltage at the terminals of the PV module varies according to the perturbations introduced by the P&O algorithm. In the test, the efficiency was over 80%. Notably, efficiency can be increased by adopting high-quality components such as SiC semiconductors [38] and techniques such as synchronous rectification, amongst others; however, in this work, experimental evaluation was performed to support the theoretical results of the conversion ratio, operation, and its potential use in PV applications.

**Figure 20:**
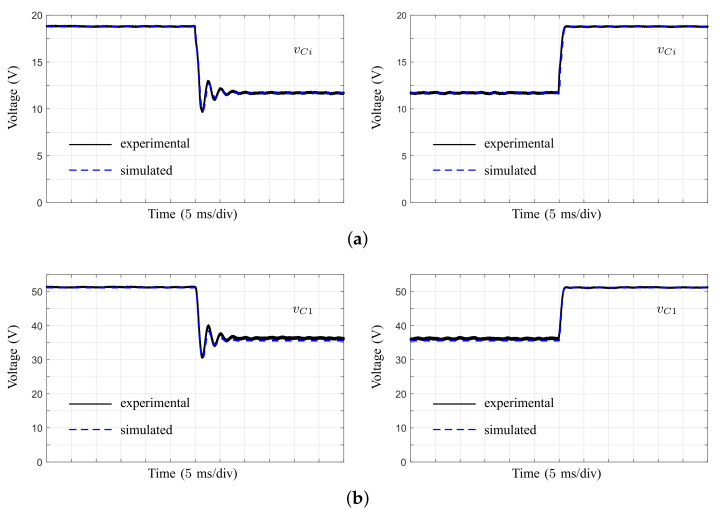
Simulation and experimental results of the transient responses of the converter under duty ratio steps. (**a**) Voltage in capacitor $C_{i}$ (time: 5 ms/div). (**b**) Voltage in capacitor $C_{1}$ (time: 5 ms/div).

Finally, the large-signal model of the converter (35) was implemented in MATLAB/Simulink (MathWorks Inc., R2021a, Natick, MA, USA), where transient responses were obtained when steps in the duty ratio were applied. Simulations and experimental results are shown in Figure 20 for capacitor voltages. In this test, the duty ratio was changed from 0.64 to 0.71 and back. As can be observed, the simulated responses predicted the dynamic response of the converter prototype, confirming the validity of the developed models.

## 5. Conclusions

This paper presented a quadratic buck-boost converter based on the interconnection of two basic switching cells in a noncascading structure, which is suitable for PV applications since the converter properly links a PV device with a DC power system. The proposed converter provides a wide voltage conversion ratio, a non-inverting output voltage, and a common node between input and output ports. The steady-state analysis demonstrated that the operation in a PV application differs when it is used in a voltage source application. Additionally, the voltage stress on semiconductors is more stringent in a PV application, whereas the current stress is low under this condition.

The experimental results showed an adequate operation of the converter under real conditions, and that the duty ratio controls the voltage at the terminals of the PV module, adjusting the generated power. The converter presents a high voltage conversion ratio, which allows wide input voltage variations with a clamped output voltage, desirable in a grid-connected applications through an inverter or microgrid applications.

## Figures and Tables

**Table 1:** Voltage and current stress on a semiconductor devices.

Semiconductor General Expression	$V_{S1}=V_{S2}$ $V_{C1}$	$V_{S3}=V_{S4}$ $V_{\mathrm{Ci}}-V_{C1}-V_{C2}$	$I_{S1}=I_{S2}$ $I_{L1}$	$I_{S3}=I_{S4}$ $I_{L2}$
Voltage converter appl	$\frac{E}{\left(1-D\right)}$	$\frac{DE}{{\left(1-D\right)}^{2}}$	$\frac{D^{3}E}{{\left(1-D\right)}^{4}R}$	$\frac{D^{2}E}{{\left(1-D\right)}^{3}R}$
PV appl–resistive load	$\frac{{\left(1-D\right)}^{3}I_{pv}R}{D^{4}}$	$\frac{{\left(1-D\right)}^{2}I_{pv}R}{D^{3}}$	$\frac{I_{pv}}{D}$	$\frac{\left(1-D\right)I_{pv}}{D^{2}}$
PV appl–clamped voltage	$\frac{V_{Ci}}{\left(1-D\right)}$	$\frac{v_{o}}{D}$	$\frac{I_{pv}}{D}$	$\frac{\left(1-D\right)I_{pv}}{D^{2}}$

**Table 2:** Main characteristics of existing quadratic buck-boost converters.

Topology	[7]	[17]	[16]	[31]	[18]	Proposed		
Source	Voltage	Voltage	Voltage	Voltage	Voltage	Voltage	PV	PV
Load	Resistive	Resistive	Resistive	Resistive	Resistive	Resistive	Resistive	Clamped
								voltage
Gain, $\frac{V_{o}}{E}$	$\frac{D^{2}}{{\left(1-D\right)}^{2}}$	$-\frac{D(2-D)}{{\left(1-D\right)}^{2}}$	$\frac{D^{2}}{{\left(1-D\right)}^{2}}$	$\frac{D^{2}}{{\left(1-D\right)}^{2}}$	$\frac{D^{2}}{{\left(1-D\right)}^{2}}$	$\frac{D^{2}}{{\left(1-D\right)}^{2}}$		
Inductors	3	2	3	2	3	2		
Capacitors	3	2	3	2	3	2		
Diodes	2	3	5	2	2	2		
Switches	2	1	1	2	2	2		
Volt. stress switches	$S_{1}:\frac{E}{{\left(1-D\right)}^{2}}$	$S_{1}:\frac{E}{{\left(1-D\right)}^{2}}$	$S_{1}:\frac{E}{{\left(1-D\right)}^{2}}$	$S_{1}:\frac{E}{\left(1-D\right)}$	$S_{1}:\frac{E}{\left(1-D\right)}$	$S_{1}:\frac{E}{\left(1-D\right)}$	$S_{1}:\frac{{\left(1-D\right)}^{3}I_{pv}R}{D^{4}}$	$S_{1}:\frac{V_{pv}}{\left(1-D\right)}$
	$S_{2}:\frac{DE}{{\left(1-D\right)}^{2}}$			$S_{2}:\frac{DE}{{\left(1-D\right)}^{2}}$	$S_{2}:\frac{DE}{{\left(1-D\right)}^{2}}$	$S_{3}:\frac{DE}{\left(1-D\right)}$	$S_{3}:\frac{{\left(1-D\right)}^{2}I_{pv}R}{D^{3}}$	$S_{3}:\frac{V_{o}}{D}$
Volt. stress diodes	$D_{1}:\frac{E}{\left(1-D\right)}$	$D_{1}:\frac{E}{\left(1-D\right)}$	$D_{1,4}:\frac{E}{\left(1-D\right)}$	$D_{1}:\frac{E}{\left(1-D\right)}$	$D_{1}:\frac{E}{\left(1-D\right)}$	$S_{2}:\frac{E}{\left(1-D\right)}$	$S_{2}:\frac{{\left(1-D\right)}^{3}I_{pv}R}{D^{4}}$	$S_{2}:\frac{V_{pv}}{\left(1-D\right)}$
	$D_{2}:\frac{DE}{{\left(1-D\right)}^{2}}$	$D_{2}:\frac{DE}{{\left(1-D\right)}^{3}}$	$D_{2,5}:\frac{DE}{{\left(1-D\right)}^{2}}$	$D_{2}:\frac{DE}{{\left(1-D\right)}^{2}}$	$D_{2}:\frac{DE}{{\left(1-D\right)}^{2}}$	$S_{4}:\frac{DE}{\left(1-D\right)}$	$S_{4}:\frac{{\left(1-D\right)}^{2}I_{pv}R}{D^{3}}$	$S_{4}:\frac{V_{o}}{D}$
		$D_{3}:\frac{E}{{\left(1-D\right)}^{2}}$	$D_{3}:\frac{E}{{\left(1-D\right)}^{2}}$					
Output polarity	Positive	Negative	Negative	Positive	Positive	Positive		
Continuous $I_{1}$	Yes	No	Yes	No	Yes	Yes		
Continuous $I_{o}$	Yes	No	Yes	No	Yes	No		
Common ground	Yes	Yes	Yes	Yes	Yes	Yes		
PV appl	Yes	Yes	No	No	Yes	Yes		

**Table 3:** Specifications of the LUXEN LNSA-160P PV module.

Characteristic	Value	Characteristic	Value
Power in MPP, $P_{pv,mpp}$	103.9 W	Open-circuit voltage, $V_{oc}$	18.86 V
Voltage in MPP, $V_{pv,mpp}$	14.01 V	Short-circuit current, $I_{sc}$	8.190 A
Current in MPP, $I_{pv,mpp}$	7.413 A		

**Table 4:** Nominal operating point of the proposed converter.

	*D*	$I_{L1}$	$I_{L2}$	$V_{C1}$	$V_{C2}$	*E*//$V_{\mathrm{Ci}}$
Voltage appl	0.6666	11.125	5.565	42.020	56	14.01
PV resistive	0.6666	11.121	5.563	42.017	56	14.01
PV clamped volt	0.6666	11.121	5.562	42.020	56	14.01

## Data Availability

Data are contained within the article.

## Abbreviations

The following abbreviations are used in this manuscript:

PV	Photovoltaic
AC	Alternating current
DC	Direct current
THD	Total harmonic distortion
MPPT	Maximum power point tracking
MPP	Maximum power point
LED	Light-emitting diode
CCM	Continuous conduction mode
DCM	Discontinuous conduction mode
P&O	Perturb and observe
